# Cardiac T_2_* mapping: Techniques and clinical applications

**DOI:** 10.1002/jmri.27023

**Published:** 2019-12-14

**Authors:** Pandji Triadyaksa, Matthijs Oudkerk, Paul E. Sijens

**Affiliations:** ^1^ University of Groningen Groningen The Netherlands; ^2^ Universitas Diponegoro, Department of Physics, Faculty of Science and Mathematics Semarang Indonesia; ^3^ Institute for Diagnostic Accuracy Groningen The Netherlands; ^4^ University Medical Center Groningen, Department of Radiology Groningen The Netherlands

**Keywords:** cardiac T2* mapping, cardiac iron overload, T2* techniques, magnetic resonance imaging

## Abstract

Cardiac T_2_* mapping is a noninvasive MRI method that is used to identify myocardial iron accumulation in several iron storage diseases such as hereditary hemochromatosis, sickle cell disease, and β‐thalassemia major. The method has improved over the years in terms of MR acquisition, focus on relative artifact‐free myocardium regions, and T_2_* quantification. Several improvement factors involved include blood pool signal suppression, the reproducibility of T_2_* measurement as affected by scanner hardware, and acquisition software. Regarding the T_2_* quantification, improvement factors include the applied curve‐fitting method with or without truncation of the signals acquired at longer echo times and whether or not T_2_* measurement focuses on multiple segmental regions or the midventricular septum only. Although already widely applied in clinical practice, data processing still differs between centers, contributing to measurement outcome variations. State of the art T_2_* measurement involves pixelwise quantification providing better spatial iron loading information than region of interest‐based quantification. Improvements have been proposed, such as on MR acquisition for free‐breathing mapping, the generation of fast mapping, noise reduction, automatic myocardial contour delineation, and different T_2_* quantification methods. This review deals with the pro and cons of different methods used to quantify T_2_* and generate T_2_* maps. The purpose is to recommend a combination of MR acquisition and T_2_* mapping quantification techniques for reliable outcomes in measuring and follow‐up of myocardial iron overload. The clinical application of cardiac T_2_* mapping for iron overload's early detection, monitoring, and treatment is addressed. The prospects of T_2_* mapping combined with different MR acquisition methods, such as cardiac T_1_ mapping, are also described.

**Level of Evidence:** 4

**Technical Efficacy Stage:** 5

J. Magn. Reson. Imaging 2019.

HEREDITARY HEMOCHROMATOSIS, sickle cell disease, and β‐thalassemia major are common blood diseases with a worldwide spread.^1^, ^2^, ^3^, ^4^, ^5^, ^6^, ^7^ Survival of the patients relies on regular blood transfusions, resulting in a high risk of cardiac failure due to iron accumulation in the heart. To prevent iron‐related heart complication during transfusion, patients receive iron chelation therapy to excrete iron from the body and thus improve life expectancy.^7^, ^8^ Effective therapy is achieved by, first, quantifying iron accumulation in the heart as a decision point to start the therapy,^3^ and, second, monitoring the outcome of the implemented iron chelation therapy regimen.^9^

Cardiac magnetic resonance imaging (MRI) provides a noninvasive method to detect cardiac iron overload by myocardial T_2_* MRI quantification. The relation between cardiac iron deposition and T_2_* value was verified by several postmortem studies.^10^, ^11^, ^12^, ^13^, ^14^ In postmortem heart samples from transfusion‐dependent anemia patients, very low cardiac MR T_2_* values were measured with confirmation of increased iron content by atomic emission spectroscopy and synchrotron x‐ray fluorescence microscopy.^10^, ^13^, ^14^ A positive curvilinear relation was found for the relation between tissue iron concentration and the R2* value in iron overload individuals.^10^ The T_2_* value detects iron overload status in blood disease patients. On its detection, T_2_* >20 msec reflects no cardiac iron overload; T_2_* between 10 and 20 msec reflects mild to moderate iron load; and T_2_* <10 msec reflects severe iron overload.^15^ Even though cardiac MR T_2_* methods have improved over the years, the pros and cons of the various methods have only been discussed briefly in clinical and technical‐scientific reports. Therefore, the roadmap for optimal application of T_2_* measurements in the detection and monitoring of myocardial iron deposition in blood diseases such as hemochromatosis and transfusion‐dependent anemia has not yet been laid out. This review discusses various aspects of T_2_* quantification proposed and in use to generate T_2_* maps. First, the technical aspects of T_2_* quantification are dealt with, including recently proposed methods for T_2_* mapping. Second, the position of T_2_* mapping in measuring iron overload in hemochromatosis and transfusion‐dependent anemia is discussed for several clinical applications, such as its early detection, heart function status, iron chelation monitoring, and its prospective combination with T_1_ mapping methods. The purpose is to recommend a combination of MR acquisition and T_2_* mapping quantification techniques for reliable outcomes in myocardial iron overload measurement and follow‐up.

## MRI Acquisition

In general, myocardial T_2_* is measured using a bright‐blood^16^ or black‐blood^17^ gradient echo sequence using a 1.5T MR scanner, with a typical acquisition given in Table 1. The T_2_* value is derived by fitting signal intensities of left ventricular (LV) myocardium regions of interests (ROIs) at different echo times (TEs) to a monoexponential equation^18^:

(1) y=Ke−TET2*

**TABLE 1:** Multigradient Echo Technique Parameters for Bright‐Blood and Black‐Blood Sequences at 1.5T

Parameters	Bright‐blood sequence	Black‐blood sequence
Minimum echo time	2 msec	2 msec
Echo time interval	2–3 msec	2–3 msec
Number of echoes	8	8
Repetition time	Depends on heart rate	Depends on heart rate
Flip angle	20^o^–35^o^	20^o^
Sampling bandwidth	810 Hz per pixel	810 Hz per pixel
Number of excitations	8	—
Cardiac gating	Yes	Yes
R‐wave triggering	Yes	Double inversion pulses
Single breath‐hold	Yes	Yes
Obtained image	10 msec after R‐wave at end‐diastolic phase	—
Inversion time	—	Extended into diastole

**FIGURE 1:**
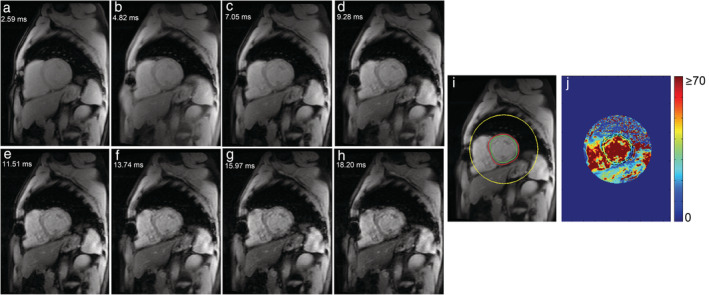
Bright‐blood multigradient echo image series **(a–h)** of midventricular short‐axis myocardium. Endocardial (green line) and epicardial (red line) contours are drawn **(i)** to represent the myocardial region (black dash lines) on the T_2_* map **(j)**.

**FIGURE 2:**
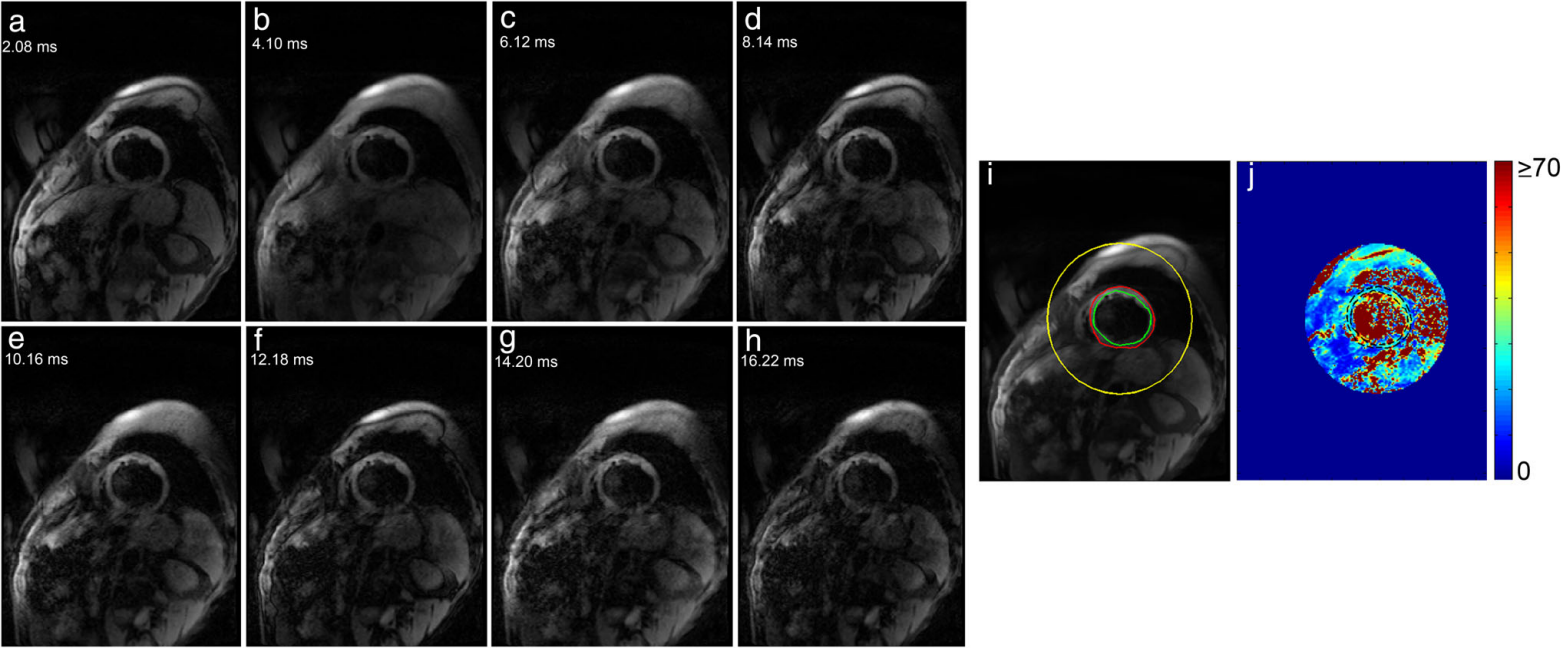
Black‐blood multigradient echo image series **(a–h)** of midventricular short‐axis myocardium. Endocardial (green line) and epicardial (red line) contours are drawn **(i)** to represent the myocardial region (black dash lines) on the T_2_* map **(j)**.

Where y represents signal intensity, K a fitting constant, TE the echo time, and T_2_* the myocardium transverse relaxation time. For acquiring a representative signal intensity of a myocardium region, epicardial and endocardial contours are drawn on a short‐axis image and propagated to all TEs (Figs. 1, 2).^16^, ^19^, ^20^ In the bright‐blood procedure, the limited contrast between the myocardium and its surroundings is a major concern inferring with accurate delineation of endocardial and epicardial borders. Therefore, the first, the second,^21^, ^22^, ^23^, ^24^ or any optimum contrast TE image^16^ has been in use as a template to draw the LV ROI. The reproducibility of T_2_* measurement was verified and validated on different scanners,^24^, ^25^ MR centers,^26^, ^27^, ^28^ and software packages,^22^, ^29^ ensuring widespread implementation^1^, ^2^, ^7^, ^30^ of the method.

### *Gradient Echo Performance at 3.0T and 7.0T*

In clinical practice, the 1.5T MR scanner has been recommended and widely used for myocardial T_2_* measurement.^31^ Nevertheless, with the increased availability of higher‐field MR equipment, further investigations have been conducted to investigate the performance of T_2_* measurement at 3.0T and 7.0T. To link the T_2_* value acquired at 3.0T to its corresponding 1.5T T_2_* value, some studies proposed a conversion relationship.^18^, ^32^ Moreover, at 7.0T, acquisition techniques were introduced to reduce the influence of the substantial macroscopic magnetic field inhomogeneities in T_2_* measurement.^33^, ^34^, ^35^ It is known that B_0_ and B_1_ inhomogeneities increase with the magnetic field strength.^36^ However, a high field benefits dynamic myocardial T_2_* mapping, better information of myocardial microstructure, visualization of myocardial fibers, detection of coronary artery stenosis, and the detection of myocardial reperfusion hemorrhage.^33^, ^34^, ^37^

Analyzing the T_2_* values at different magnetic field strengths showed roughly halved myocardial T_2_* value when comparing its linear correlation at 3.0T to 1.5T in iron‐overload patients.^18^, ^32^ Compared with 3.0T, the performance of T_2_* measurement at 1.5T showed a good intraobserver and interobserver agreement with lower variability, both when using bright‐blood and black‐blood multigradient echo (MGE) sequences.^36^ It is important to note that the increase of magnetic field strength changes the location of susceptibility artifacts in the myocardium. At 3.0T, basal inferolateral wall, basal inferior wall, and midventricular inferior wall are most affected by the artifacts. Furthermore, at 7.0T the inferolateral wall is the most affected region by the artifacts.^37^, ^38^ Reference T_2_* values to determine iron status at 1.5T and 3.0T are tabulated in Table 2.^32^, ^39^

**TABLE 2:** Cardiac MRI Reference Values for Myocardial Iron Status at Different Magnetic Field Strengths

Iron loading stratification	Cardiac MRI 1.5T		Cardiac MRI 3T	
	T2* (msec)	R2* (Hz)	T2* (msec)	R2* (Hz)
Normal	>20	<50	>12	<83
Moderate	10–20	50–100	5.5–12	83–181
Severe	<10	>100	<5.5	>181

### *Bright‐Blood Gradient Echo*

In bright‐blood gradient echo for T_2_* assessment, a single breath‐hold technique with electrocardiogram‐gating is used to acquire multiple TE images by the MGE sequence.^22^, ^40^ The total acquisition time in a single breath‐hold technique depends on the heart rate.^19^, ^20^, ^22^ It has been suggested to incorporate some delay, ie, 10 msec, after the R‐wave at the end‐diastolic phase to obtain images avoiding heart motion artifact.^17^, ^19^, ^21^ For patients having difficulties maintaining breath‐hold, it is advised to increase the number of excitations.^41^ Flip angle in this technique is typically set between 20°–35°.^19^, ^20^, ^21^, ^24^, ^25^, ^26^, ^28^, ^40^, ^42^, ^43^ When poor breath‐holding becomes a problem, such as for the cardiac arrhythmia patient, free‐breathing T_2_* measurement has been an alternative, even though used sparingly up to now.^44^, ^45^

The experiment showed that the influence of water and fat protons at the first out‐of‐phase and in‐phase TE points is minimized when the first TE was set near to 1 msec. This setting yields more accurate T_2_* measurements when compared with synthetic T_2_* values.^41^ However, for a minimum TE of 2 msec, a biased estimation is expected for measured T_2_* values under 3 msec.^41^ The MGE series is preferred to acquire images at 8–10 different TEs at 2–3‐msec intervals.^32^, ^41^, ^46^ A consensus statement in cardiac MR mapping suggested ranging the TE from 2–18 msec at 1.5T.^31^ At longer TE, the noise level was found to mainly result in a lower signal‐to‐noise ratio (SNR) for a lower number of excitations.^12^

### *Artifacts*

**FIGURE 3:**
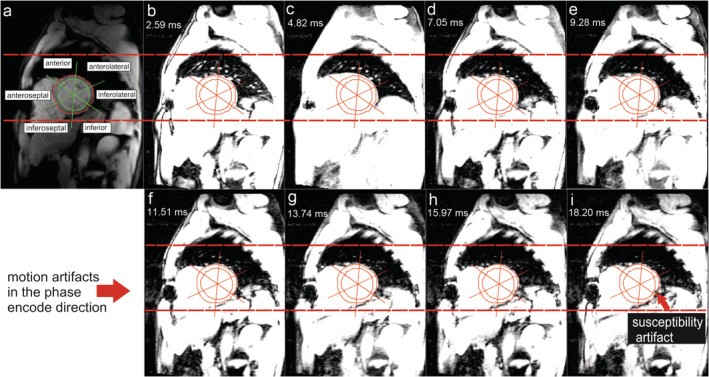
Artifacts appearance on a bright‐blood multigradient echo image series. At midventricular short‐axis myocardium **(a)**, a specific window level and window width setting of Fig. 1 enhances the presence of motion artifacts propagated in the phase‐encode direction (located by dash lines) and susceptibility artifact progression at inferior and inferolateral segments of the left ventricle **(b–i)**.

When applying the bright‐blood MGE at 1.5T MRI, artifacts tend to appear at different TE images of the myocardium (Fig. 3), which may lead to overestimation of the amount of iron deposited in the myocardium. The influence of large cardiac veins such as the great cardiac vein close to the anterior wall, the middle cardiac vein, and the posterior vein close to the inferior wall of LV myocardium contribute to the perturbation of the z‐component of the magnetic field. The perturbation occurs due to susceptibility differences between the surrounding tissues and the deoxygenated blood in the vein. The effect of this artifact drops fast with increasing distance between the LV region and the source. Different types of the artifact can also appear due to the heart–lung interface at the lateral wall, epicardial fat at the anterior wall, or cardiac motion artifact.^20^, ^23^, ^44^, ^47^

Susceptibility artifact was reported to mainly influence myocardial T_2_* measurement at a higher global T_2_* value (ie, greater than 32 msec) and become negligible in heavily iron‐loaded myocardium^21^, ^48^ or region‐based T_2_* measurement method.^49^ Extending the longest TE of the MGE sequence also increases distortion in the phase‐encoding direction, leading to degraded short‐axis image quality because of severe field inhomogeneities.^47^ It is important to note that the intensity of susceptibility artifact mainly depends on the angle between the axis of the vein and the acquired slice. Therefore, the presence of artifacts may vary between different patient examinations.^20^, ^48^ Different quantitative methods have been proposed to detect or correct artifact in T_2_* measurement.^20^, ^23^, ^44^, ^45^, ^48^

A single breath‐hold with electrocardiogram‐triggered MGE is commonly used to reduce the influence of motion artifact. However, in a case when the patient is unable to perform the breath‐hold, another method such as a free‐breathing method can be an alternative. An example is a single‐shot gradient‐echo echo‐planar‐imaging (GRE EPI) sequence combined with a nonrigid motion correction.^44^, ^45^ The method works by acquiring a single image per TE on each heartbeat, which may be accelerated by using a segmented EPI readout. It is known that EPI enables fast image acquisition and the elimination of motion‐related artifacts.^50^

In MRI, signal intensity alteration due to different proton precession resonance frequencies can induce a chemical shift phenomenon. This induction causes spatial misregistration artifact on image data along the frequency‐encoded direction.^51^ The artifact is evident between water and lipid signals due to its relatively significant differences in magnetic shielding. The effect of the misregistration can be seen as a dark or bright band of signal intensity at the interface between lipid and water in the frequency‐encoded direction of the image. The band occurs when the misregistration is greater than the individual pixel size. It is important to note that the selection of frequency‐encoding direction mostly influences the size and location of the chemical shift artifact.

The effect of chemical shift artifact can be minimized by implementing fat‐suppression techniques such as spectral saturation or by setting a specific MR sequence setup such as frequency‐encoding direction, the field of view (FOV), and receiver bandwidth.^51^ The minimum effect of the artifact is achieved by using a smaller FOV and by avoiding narrow receiver bandwidth of less than ±16 kHz. The relation between the frequency shift, FOV, and bandwidth in creating chemical shift distance is explained as follows^51^:

(2) $$\text{Chemical shift distance}=\frac{\text{Frequency shift}\left(\mathrm{Hz}\right)\times \text{Field of view}\;\left(\mathrm{mm}\right)}{\text{Bandwidth}\;\left(\mathrm{Hz}\right)}$$

The application of less than 180° radiofrequency pulse in gradient echo sequences influences the in‐phase and out‐of‐phase condition between water photon and fat signals over time. At 1.5T, the in‐phase precession signal of every ±4.4 msec between fat and water gives an additional signal contribution to the resultant image, while at its out‐of‐phase condition, the resultant image will receive the contribution of fat and water signal differences.^51^

### *Black‐Blood Gradient Echo*

Another method to generate a series of MGE images is known as the black‐blood sequence.^17^ Here the blood pool signal is suppressed by double inversion pulses at the R‐wave trigger, with the setting of the inversion time extended into diastole. The TE images are acquired in a single breath‐hold, with the set of flip angles, shortest TE, TE intervals, and the number of collected TE images similar to the corresponding parameters in the bright‐blood sequence. As a result, a more homogenous myocardium region is obtained with good contrast between the myocardium and its surroundings, such as the blood pool and lung. The improvement of contrast difference, therefore, provides better image information for the delineation of endocardial and epicardial borders. The method also reduces susceptibility artifact in myocardium regions close to large cardiac veins.^17^, ^52^, ^53^, ^54^

Application of the black‐blood MGE sequence in T_2_* measurement has been shown to improve image quality, the goodness of monoexponential curve fitting, and intraobserver and interobserver reproducibility compared with the use of bright‐blood MGE.^17^, ^52^, ^53^, ^54^ Study comparisons between the two sequences also revealed a good agreement of T_2_* values measured by the two with higher T_2_* reproducibility when using the black‐blood MGE sequence.^42^, ^52^, ^53^, ^54^ Therefore, black‐blood MGE is recommended for clinical routine T_2_* measurement when available.^31^

## T_2_* Mapping Techniques

### *Myocardium ROI Selection*

**FIGURE 4:**
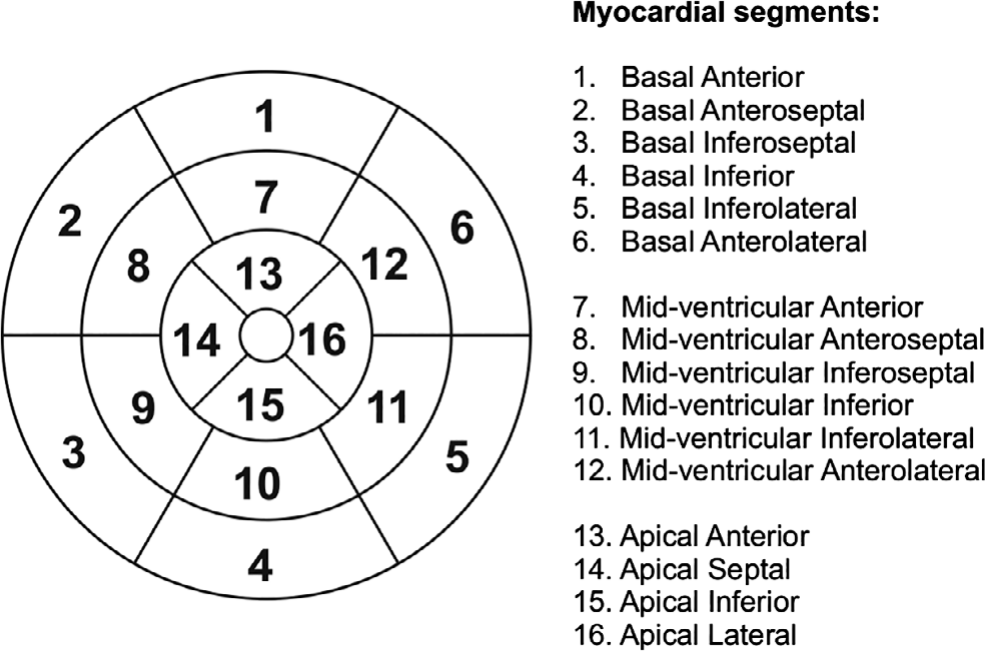
Bull's‐eye plots showing global myocardium using AHA 16‐segments model where midventricular septum approximates the sum of segments 8 and 9.

The entire midventricular septum is used to measure iron in LV myocardium,^3^, ^31^, ^55^, ^56^ while others prefer to measure global iron myocardium per segment using American Heart Association (AHA) 16‐segment model (Fig. 4).^25^, ^43^, ^46^, ^57^ Iron distribution identification in postmortem heart samples with severe iron overload revealed higher iron concentration in the epicardial than in the endocardial region.^10^, ^11^, ^13^ It has been recommended to use midventricular septum as a comparatively artifact‐free region to measure iron overload.^31^ The postmortem finding showed that iron distribution is heterogeneous in the myocardium, as reported using the global longitudinal strain method,^43^ and was confirmed by the T_2_* measurements in global myocardium.^19^, ^20^, ^21^, ^25^, ^27^, ^41^, ^43^, ^45^, ^49^, ^57^, ^58^, ^59^ Different iron overload patterns in different myocardial segments beyond the influence of susceptibility artifact were observed. The observation is evident, especially in those studies where the presence of artifacts due to susceptibility and heart motion were corrected for.^20^, ^44^, ^45^, ^48^ In detecting early progression of iron overload, global myocardium characterization per segment thus gives more information on iron deposition heterogeneity than can be derived from the midventricular septum region.^57^, ^58^, ^59^ Therefore, iron monitoring in global myocardium is beneficial to detect early iron loading, in particular in pediatric patients.^58^, ^60^ Future challenges remain to confirm the pattern of iron progression, which has been described in the above‐mentioned postmortem studies.

### T_2_* Mapping Methods

In general, there are two ways to calculate T_2_* in the ROI of LV myocardium. The first method, known as ROI‐based, averages signal intensity in the ROI on each MGE image before fitting its value monoexponentially, while the other so‐called pixelwise method fits the signal intensity of each pixel in the ROI of MGE image series separately. Both methods were reported to perform similarly in measuring the T_2_* value of normal and moderate iron status on a synthetic T_2_* map.^41^, ^42^, ^53^ However, for T_2_* <10 msec the pixelwise method can identify more precisely the correct T_2_* value than the ROI‐based method.^41^ The variability of T_2_* measurement by the pixelwise method has also been reported to be small compared with the ROI‐based method.^61^, ^62^

The pixelwise method has the benefit of detecting iron heterogeneity across myocardial regions both on a bright‐blood MGE sequence (Fig. 1j) and a black‐blood MGE sequence (Fig. 2j). A more accurate T_2_* measurement by the pixelwise method can be achieved by evaluating the median of pixelwise T_2_* rather than its mean value yielding higher T_2_* measurement reproducibility.^46^, ^61^, ^62^ Triadyaksa et al reported that the median pixelwise method could be used regardless of any statistical normality distribution of the data.^63^ In early detection of heart function abnormality, the median pixelwise method has shown sensitivity for identifying heart function impairment, which was undetected by using the mean.^63^ Misinterpreted T_2_* value distributions can lead to misleading iron deposition status, interfering with the early detection of iron loading in borderline T_2_* threshold situations, affecting the decision for appropriate chelation therapy.

Noise bias reportedly reduces the accuracy and precision of the pixelwise method and is related to the number of MR receiver coil elements used.^64^ To compensate for the effect of noise bias, a standard deviation map was proposed to evaluate the quality of the pixelwise T_2_* measurement.^64^ The proposed map can also evaluate the performance of the T_2_* imaging protocol, ie, by using parallel imaging acceleration to identify any random error in the image while failing to identify any systematic bias due to the susceptibility of gradients.

**FIGURE 5:**
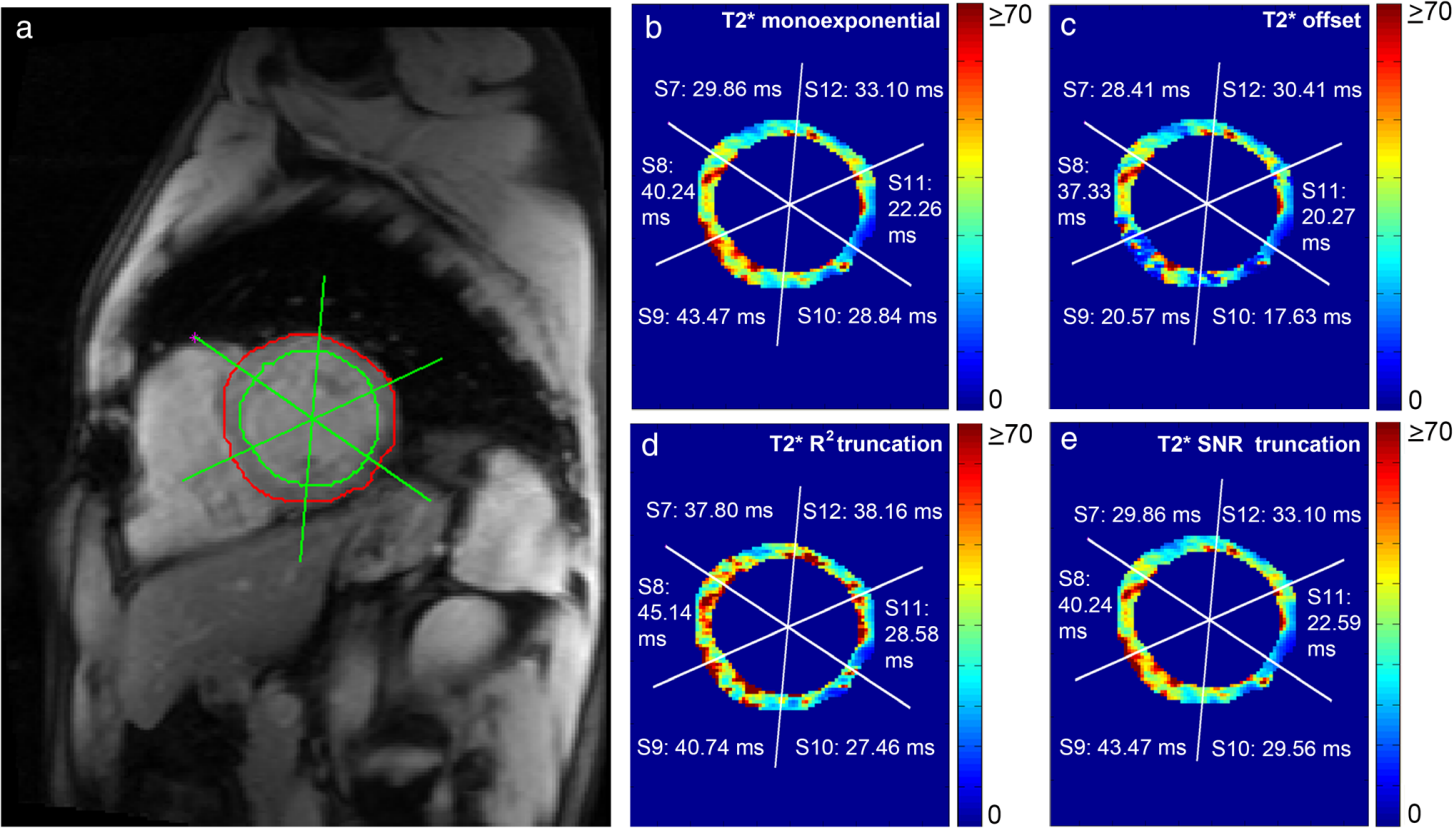
Pixelwise monoexponential T_2_* fitting of full left midventricular myocardium image **(a)** without **(b)** and with an offset **(c)** or truncation based on R^2^ **(d)** or SNR **(e)**, with the same T_2_* range, performed differently by handling the presence of motion artifacts as described in Fig. 3 yielding different segmental T_2_* values.

**FIGURE 6:**
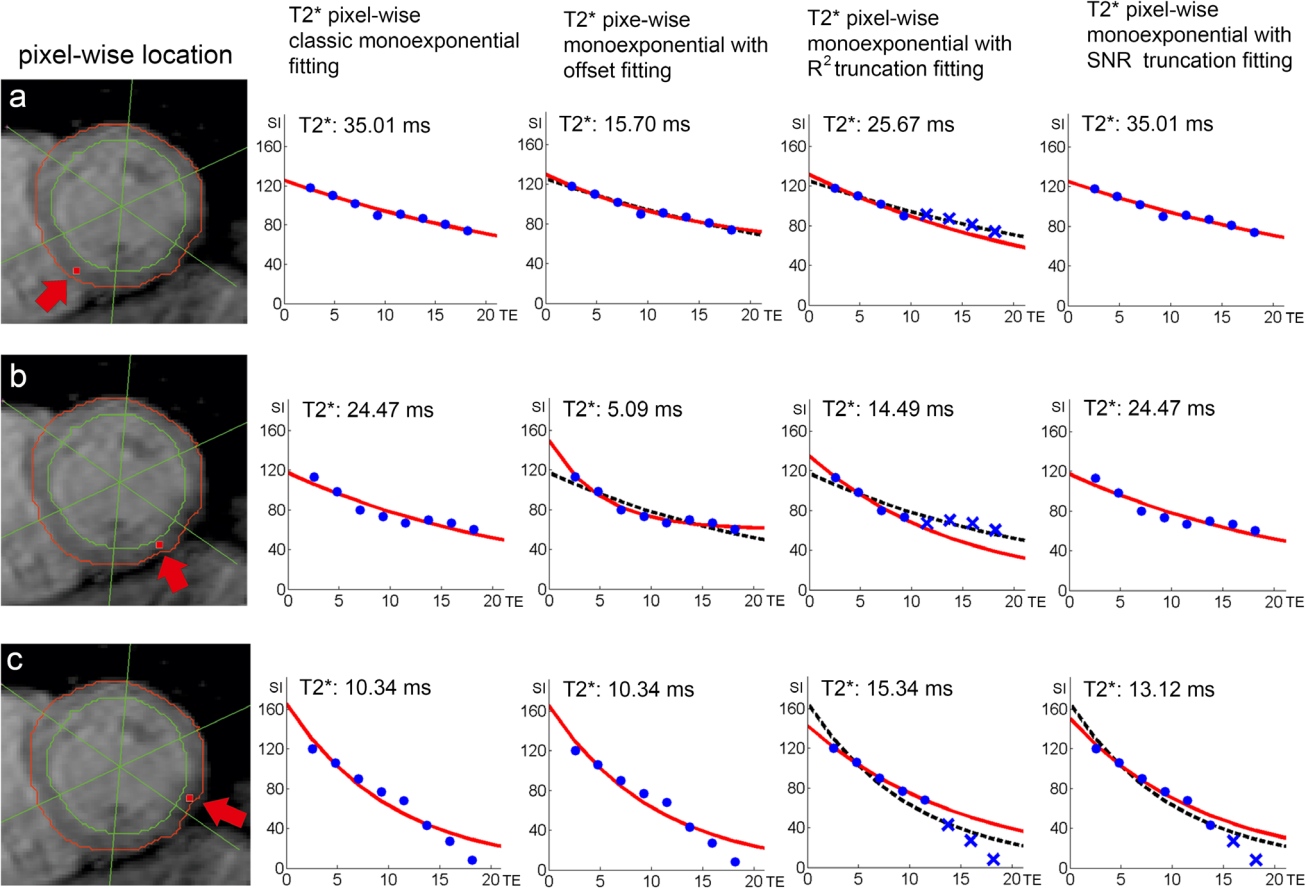
Pixelwise T_2_* measurement depicted at inferoseptal **(a)**, inferior **(b)** and inferolateral **(c)** on left midventricular short‐axis myocardia as shown by arrowheads. The monoexponential fitting plots (red line) of the four methods correspond to the locations in the presence of motion artifacts at the phase‐encode direction and susceptibility artifact highlighted in Fig. 3. The dashed line on the fitting plots represents classic monoexponential fitting of the respective alternative methods.

Due to the presence of signal plateau or offset at longer TEs, different modifications of monoexponential fitting were introduced to acquired more reliable T_2_* values (Figs. 5, 6). A modification by adding an offset constant to the classic monoexponential equation (Eq. 1), further known as the monoexponential offset method, was proposed^65^ (Fig. 5c):

(3) y=Ke−TET2*+C

with y, K, TE, T_2_* as in Eq. 1 and C representing a constant of offset correction. Another way of dealing with the signal plateau is by applying a truncation at longer TEs known as the R^2^ monoexponential truncation method (Fig. 5d). The method evaluates the goodness of the monoexponential fit (R^2^) as a criterion to exclude data points at longer TEs. In this method, different R^2^ values of 0.990,^13^, ^42^ 0.995,^66^, ^67^, ^68^ or fitting error threshold of 5%^46^ have been used. Another truncation method involves the exclusion of any data point at longer TEs with SNR below the noise level of the monoexponential fit. This method is known as the SNR monoexponential truncation method (Fig. 5e). The method works by excluding any data point at longer TEs from the monoexponential fit, having SNR below the noise level. Different SNR thresholds were adopted in discharging data points with confirmed SNR of below 2^12^ or 2.5.^64^ Automatic truncation procedures to generate T2* maps were proposed in several studies by either SNR^64^ or R^2^ method.^46^, ^68^

A comparison between the offset method and the truncation method shows that lower T_2_* values are produced by the offset method by using the lower number of excitations.^12^ Studies also reported an underestimation of T_2_* value by the offset with the decrease of "true" model T_2_*, while both SNR and R^2^ truncation methods were able to fit the model properly until lower T_2_*, confirming higher accuracy and precision of the truncation methods in measuring T_2_*.^67^, ^69^ The illustrations in Figs. 5 and 6 also demonstrate a lower T_2_* measurement by the offset method compared with other monoexponential fitting methods in the presence of artifacts of a real patient's data. However, to our knowledge, the two truncation methods were not compared in the presence of major susceptibility or motion artifacts. Moreover, different thresholds in excluding longer TE points by the two methods also need to be validated for more accurate and precise T_2_* measurement.

### *Automatic LV Myocardium Definition*

The delineation technique to determine the myocardial region plays an essential role in detecting iron overload in the whole myocardial area while avoiding any adjacent regions, ie, blood pool, lung, or stomach. Manual delineation of myocardial borders necessarily produces variability between observers that decrease with higher cardiac imaging experience.^16^, ^65^ To reduce observer's subjective variability, several studies proposed automatic LV myocardial segmentation on bright‐blood^65^, ^70^ and black‐blood^71^, ^72^ gradient echo sequences with promising results to replacing manual delineation. Due to the different methods to acquire gradient echo images by the two sequences, different segmentation approaches are needed. In the black‐blood image, better contrast between myocardium and LV blood pool allows effective implementation of image thresholding^72^ or Hough transformation^71^ methods to initiate the location of the LV blood pool, continued by implementing region‐growing or active contour methods. In the bright‐blood image, however, due to lower contrast between myocardium and LV blood pool, k‐means clustering on low signal intensity blood pool^65^ or morphological operations^70^ are needed to define the prospective LV location and continued by adapted active contour methods.

## Clinical Application of T_2_* Mapping

### *Early Diagnosis of Myocardial Iron Overload*

Carpenter et al reported a worldwide survey of cardiac MR T_2_* that shows a moderate or severe cardiac iron overload condition at initial cardiac MR scanning.^1^ To avoid this condition, early detection of iron loading by T_2_* measurement is needed and followed by an appropriate iron chelation therapy to improve the survival rate of hemochromatosis and transfusion‐dependent anemia patients.^15^, ^30^, ^73^, ^74^ Several studies reported a reduction of age recommendation to start the first T_2_* screening for thalassemia major patients from 10 years old to as early as 5 years old. The reduction promotes early detection of iron loading in pediatric patients.^2^, ^3^, ^9^, ^58^, ^75^ The screening was proposed to respond to suboptimal iron chelation therapy that can induce iron accumulation at an early age. The detection of iron accumulation at an early age is still important to avoid later liver and cardiac impairment, despite reports showing later accumulation of myocardial iron after a more extended transfusion period in sickle cell disease and myelodysplastic syndrome.^76^, ^77^ Chouliaras et al reported the use of the first cardiac MR T_2_* scanning as additional decision information for iron chelation therapy regimens, showing that the information works to drastically reduce cardiac‐related death by 45% compared with patients lacking the information on iron content.^78^

### *Myocardial Function Status in Iron Overload Conditions*

The monitoring of changes in functional cardiac MR and echocardiography can detect the progression of heart function impairment due to iron loading. The normal range value of LV function in thalassemia major patients without myocardial iron overload has been documented^79^, ^80^ and reported to differ from LV ejection fraction (LVEF) normal values in healthy individuals.^81^ When observed in hemochromatosis and transfusion‐dependent anemia patients, a decrease of LV, and right ventricle (RV) ejection fraction was found with an increase of cardiac iron accumulation, as confirmed by T_2_* <20 msec.^59^, ^82^, ^83^, ^84^, ^85^ In these cases, septal thickness, LV mass index, LV end‐systolic volume (ESV), and RV ESV were increased.^56^, ^82^, ^83^, ^84^, ^85^ Nevertheless, contradicting LV end‐diastolic volume values were reported by several studies observing no significant differences between patients and controls.^56^, ^79^, ^84^, ^85^ A decrease of RV and LV stroke volume was found in patients with increased cardiac iron.^59^ In beta‐thalassemia major patients with significant iron accumulation, echocardiography analysis showed a reduction of left atria (LA) peak positive strain, LA total strain, right atria (RA) peak positive strain, RA total strain, LV global longitudinal strain, and LV global circumferential strain.^43^, ^86^

### *Effective Iron Chelation Therapy Monitoring*

In hemochromatosis and transfusion‐dependent anemia patients, the detection of the T_2_* value lower than 10 msec indicates a high‐risk development of heart failure.^3^, ^87^ Iron chelation therapy is used to prevent this condition by removing iron from cells and balance the body iron to its normal level, especially for patients receiving a long‐term transfusion. To prevent this condition, especially for patients receiving a long‐term transfusion, iron chelation therapy is used to remove iron from cells and balance the body iron to its normal level. Ideally, the therapy should be initiated at an early age before the significant occurrence of iron accumulation.^3^, ^88^ Studies showed that suboptimal or minimum access to regular chelation therapy could induce cardiac complications^6^, ^15^, ^30^ and increase the cost of cardiac iron overload treatment.^89^ Several chelating agents are used to excrete iron in the body, such as deferoxamine, deferasirox, and deferiprone, with a reportedly prescribed dose.^88^ The pros and cons of monotherapy or combined therapy of the chelating agents were reported by recent studies,^4^, ^5^, ^6^, ^8^, ^73^, ^74^ promoting caution in chelation monitoring to control its side effects. Because of differences in chelation therapy practice between countries and regions,^2^ monitoring of any complication effect of the therapy should be performed regularly according to a schedule proposed recently. Iron overload monitoring is advisedly performed every 6 months when T_2_* values are below 10 msec.^9^ Cardiac MR T_2_* measurement thus plays a vital role in updating the patient's iron status during the chelation therapy monitoring,^2^, ^4^, ^5^, ^9^, ^73^, ^74^ resulting in more accurate and effective iron chelation regimens for transfusion‐dependent anemia patients.

### *Complementary Techniques Used for Cardiac Assessment*

Improvement of LV function such as ejection fraction can be obtained by iron‐chelation therapy, leading to prolonged survival age and reduction of death rate by cardiac failure. However, when the therapy fails to improve the LV function, or there is no access to regular therapy,^3^, ^15^, ^75^, ^89^, ^90^ LV impairment becomes worse and leads to heart failure. The progression of LV impairment can thus be detected independent of heart iron^81^ and also by the presence of myocardial fibrosis.^30^, ^91^ The quantification of myocardial extracellular volume (ECV) by cardiac MR shows higher ECV fraction in thalassemia major patients undergoing chelation therapy, indicating the presence of myocardial fibrosis.^92^ Although interstitial fibrosis has been demonstrated in thalassemic patients,^30^, ^93^ the use of ECV for detecting diffuse fibrosis could be a more sensitive parameter. T_2_* mapping is able to differentiate patients with and without myocardial fibrosis in hypertrophic cardiomyopathy patients,^56^ while in acute myocardial infarction patients, T_2_* mapping values under 20 msec are related to the presence of microvascular injury.^94^

Lower T_1_ value, compared with controls, is identified in transfusion‐dependent anemia patients having a T_2_* value under 20 msec.^55^, ^95^ Krittayaphong et al proposed a cutoff native T_1_ mapping value of 900 msec by using the modified Look–Locker inversion recovery sequence (MOLLI) at 1.5T MRI in thalassemia patients with cardiac iron overload,^96^ not unlike the thresholds reported in other studies.^97^, ^98^ When using the shortened modified Look–Locker inversion recovery sequence, the T_1_ value was slightly lower compared with MOLLI.^95^ T_1_ measurement in the case of cardiac iron overload at 3T revealed a modest increase of T_1_ value compared with the value measured at 1.5T. However, the measured T_1_ value is still lower compared with the controls measured at that field strength.^97^, ^98^ A challenge remains to validate the correlation of myocardial iron concentration and fibrosis to T_1_ mapping in postmortem studies.^14^, ^99^ Nevertheless, the values of T_1_ mapping and ECV measurement can be used as complementary information of T_2_* mapping to identify LV impairment and iron status in cardiac iron overload patients.

## Recommendation and Prospect in Iron Overload Detection

In conclusion, we recommend several techniques to generate T_2_* mapping in myocardial iron overload measurement. The application of a black‐blood gradient echo sequence to generate pixelwise T_2_* mapping is recommended to improve the contrast between myocardium and its surroundings and to remove any blood signals inducing susceptibility artifact. A standard deviation map can be used as additional information to detect the presence of artifacts in the T_2_* map. Image series should be acquired in eight TEs ranging from 1–18 msec at recommended 1.5T. In bright‐blood gradient echo sequence, some delay after the R‐wave at the end‐diastolic phase is suggested to avoid the heart motion artifact with advisedly increasing the number of excitations for patients having difficulties maintaining breath‐hold. Free‐breathing gradient‐echo using single‐shot GRE EPI with a respiratory motion correction can also be an alternative technique to produce higher quality of the T_2_* map when the reproducibility can be validated at different MR centers and scanners.

Due to its ability to suppress artifact, the use of a black‐blood sequence facilitates iron heterogeneity identification on different AHA segments for early detection of myocardial iron overload and chelation treatment monitoring. In order to identify iron heterogeneity on the T_2_* map, the median assessment is proposed to quantify its pixelwise regional values. Automatic segmentation of LV myocardium can serve as an alternative method to gain higher reproducibility in myocardial region definition, and thus improves accurate iron overload detection. A modification of monoexponential fitting with truncating longer TE in the presence of signal plateau was shown to produce more reliable T_2_* measurement with the need for further validation of the methods in detecting true iron in the presence of unwanted artifact. Automatic truncation in SNR and R^2^ truncation methods potentially serves as an objective technique to exclude longer TE data in T_2_* map generation. The presence of iron overload is recommended to be confirmed by other techniques, such as T_1_ mapping and ECV quantification, for a better understanding of the progression of the disease.
